# Draft genome sequences of *Salmonella* Oslo isolated from seafood and its laboratory generated auxotrophic mutant

**DOI:** 10.7150/jgen.40059

**Published:** 2020-01-01

**Authors:** Kadeeja Jazeela, Anirban Chakraborty, Praveen Rai, Ballamoole Krishna Kumar, Shabarinath Srikumar, Scot van Nguyen, Daniel Hurley, Seamus Fanning, Indrani Karunasagar, Vijaya Kumar Deekshit

**Affiliations:** 1 Nitte University Center for Science Education and Research, Nitte (Deemed to be University), Deralakatte, Mangaluru - 575018, Karnataka, India.; 2 University College Dublin, Food Safety and zoonoses, Dublin, Ireland.

**Keywords:** Non typhoidal *Salmonella*, * Salmonella* Oslo, Bacteria mediated cancer therapy, whole genome sequence

## Abstract

In recent years, the concept of bacteria-mediated cancer therapy has gained significant attention as an alternative to conventional therapy. The focus has been on non-typhoidal *Salmonella* (NTS), particularly *S.* Typhimurium, for its anti-cancer properties, however, other NTS serovars such as *Salmonella* Oslo, which are associated with foodborne illnesses could potentially be effective anti-cancer agents. Here, we report the draft genome sequence of *Salmonella* Oslo isolated from seafood and its laboratory generated auxotrophic mutant.

## Introduction

Non-typhoidal *Salmonella* (NTS) is one of the pathogens that frequently cause foodborne infections throughout the world. The pathogenic potential of NTS strains have been well understood 1. However, its therapeutic potential as an anti-cancer agent remain unexplored. Among more than 2500 NTS serovars, *S*. Typhimurium VNP20009 2-4, an auxotrophic mutant strain of *S*. Typhimurium A1-R 5, 6, has been successfully studied and was even tested in phase I clinical trial for the treatment of solid tumors 7. Many features of *Salmonella* namely the ability to thrive in hypoxic environment of the tumor, to induce innate immune response against tumor or its bactofection, and ability to release anticancer genes within tumor were found to be favorable for tumor regression in animal models 8-10. However, despite the success observed in animal models, the clinical trials didn't yield expected results. This prompted the researchers to look for alternative NTS strains for their anticancer properties. In this study, the aim was to sequence the genomes of *Salmonella* Oslo isolated from seafood and its laboratory generated auxotrophic mutant. The availability of genetic information provides a basis for further studies, particularly for investigating their role as anti-cancer agents.

*Salmonella* Oslo was isolated from seafood (squid sample) as per the protocol recommended by the FDA Bacteriological Analytical Manual 11 with minor modifications. Briefly, the seafood sample was pre-enriched in lactose broth, followed by enrichment in selenite cysteine broth and tetrathionate broth. Post enrichment, the sample was streaked on Hektoen enteric agar (HiMedia Laboratory Pvt Ltd, India). During the enrichment and plating steps, the incubation temperature was maintained at 37º C for 16 to 18 h. Colonies with specific morphological features were selected and were subjected to a series of biochemical tests such as indole test, methyl red test, Voges-Proskuer test, citrate test, triple sugar iron agar (TSIA) test, urease and lysine iron agar (LIA) test for conventional identification. The biochemically positive colonies were further confirmed by PCR using genus-specific primer *invA* 12. Serotyping was done at National *Salmonella* and *Escherichia* Centre, Central Research Institute, Kasauli, India.

Lambda red recombinase method 13 was used to generate the auxotrophic mutant of *Salmonella* Oslo by inducing deletions in *argH* and *leuB* genes, which code for arginine and leucine respectively. Biofilm assay was performed using the method described by Stepanovic et al (2004) 14.

To compare the growth kinetics of *Salmonella* Oslo (SO1-wild type) and its mutant (LAT9), a 100 *µ*l aliquot of overnight culture of SO1 and LAT9 was added to 5 ml Luria Bertani broth (HiMedia Laboratory Pvt Ltd, India) and incubated at 37°C with shaking at 200 rpm. The optical density was measured at 600 nm (OD_600_) at different time points such as 0, 1, 2 to 24 h after incubation and expressed as log (OD_600_ X 1000).

To sequence the genome of SO1 and its laboratory generated mutant, bacterial genomic DNA was extracted using a QIAamp DNA mini kit (Qiagen, Germany). The quality of the extracted DNA was checked by Qubit^®^ and further verified by a bioanalyzer (Agilent technologies). The genomic DNA library was prepared using a Nextera XT DNA library preparation kit (Illumina, Inc, Cambridge, UK). The whole genome sequencing was performed at UCD, Dublin. The raw sequence data were generated using the Illumina MiSeq platform with a depth of 100x. The obtained paired end reads were merged and the genome was assembled using CLC genomics (version 11) 15. The processed reads were aligned to the reference genome LT2 strain (LT571437) with Bowtie2 program. The annotation and gene prediction of draft genome was done using the Rapid Annotations Subsystems Technology (RAST) (http://rast.nmpdr.org/) 16.

**Figure 1:**
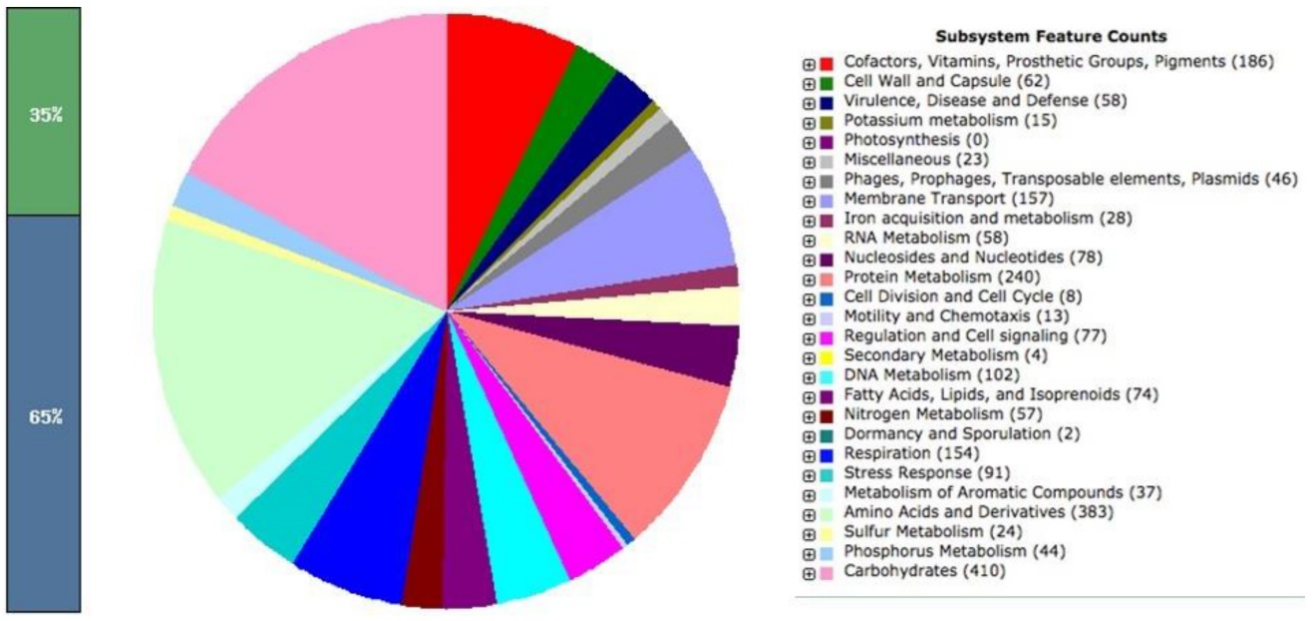
* Salmonella Oslo* (SO1)-WT subsystem feature.

The identification of the isolate as true *Salmonella enterica* serovar Oslo was confirmed by serotyping experiments. As expected, the auxotrophic strain (LAT9) was found to be phenotypically mutant to amino acid arginine and leucine. However, when PCR was performed to confirm the changes in the target genes (*argH* and *leuB*), it revealed no deletions in *argH* and *leuB* genes. This strange observation prompted us to determine the whole genome sequence of wild (SO1) and mutant (LAT9) strain. The generated library produced a total of 1193762 and 1221694 reads for wild type and mutant respectively. The paired end reads of SO1 were assembled into 121 contigs with coverage of 100x. The genome size was calculated at 4,860,262 bp comprising of 4,974 protein coding genes. The GC content of this strain was found to be 52.2%. The analysis obtained from the RAST also revealed 401 subsystems (Fig. 1). The annotated genome had 383 amino acid biosynthesis genes including *argH* and *leuB*. In addition, 78 tRNAs, 11 ncRNAs, 168 pseudo genes were also identified.

**Figure 2:**
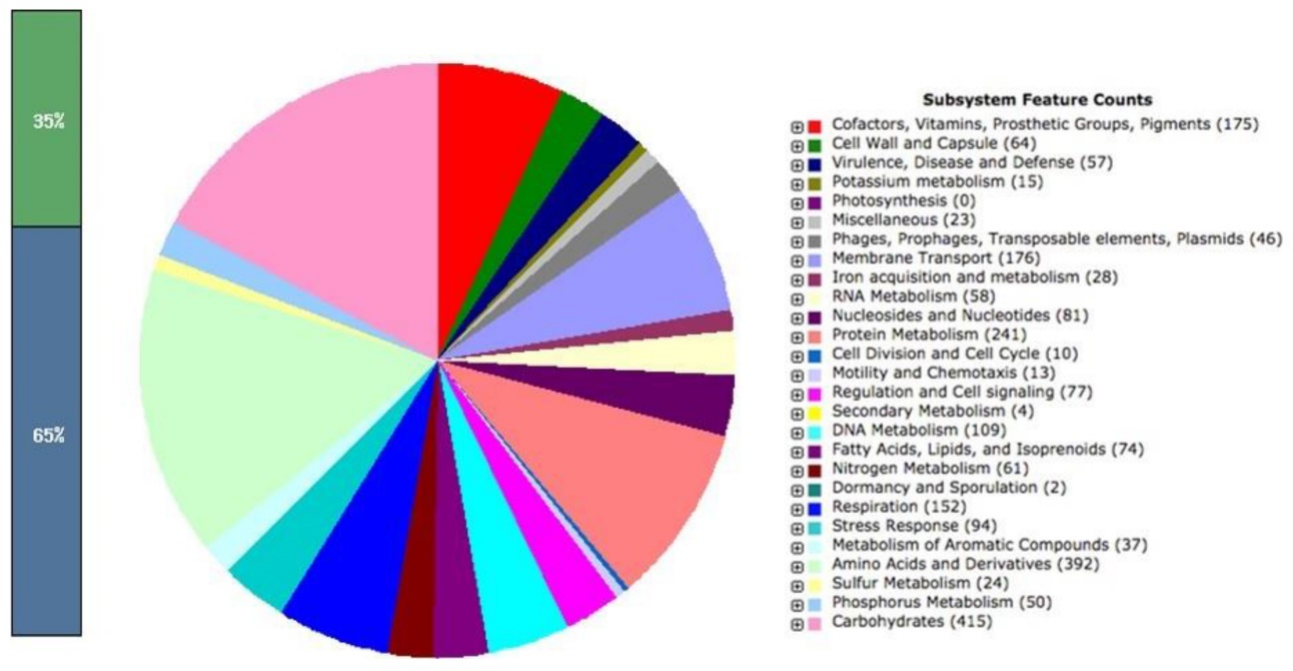
*Salmonella* Oslo (LAT9) subsystem features.

Similarly, the paired end reads of LAT9 were assembled into 199 contigs with coverage of 100x. The genome size was calculated at 4,890,414 bp comprising of 5,082 protein coding genes. The GC content of this strain was found to be 52.2%. The analysis obtained from the RAST also revealed 402 subsystems (Fig. 2). The annotated genome had 392 amino acid biosynthesis genes. 79 tRNAs, 11 ncRNAs, 239 pseudo genes were also identified.

**Figure 3:**
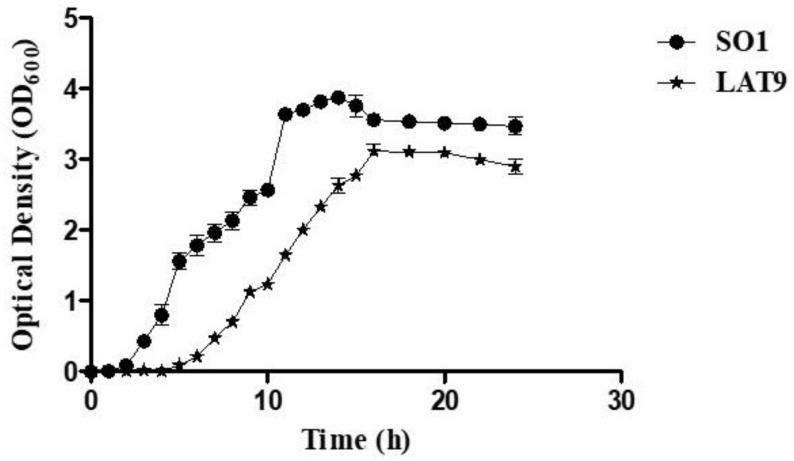
Growth kinetics of SO1 and LAT9.

**Figure 4:**
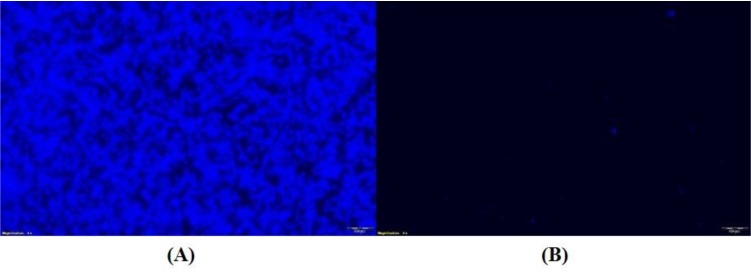
Calcoflour stained images of SO1 (A) and LAT9 (B) strains under fluorescent microscope.

Further, the growth kinetic analysis revealed that the growth of LAT9 was significantly (*p-*value of >0.001) slower than SO1 (Fig 3). The biofilm forming ability of LAT9 was also significantly reduced when compared with SO1 (Fig 4).

To the best of our knowledge, this is the first draft genome sequence of *Salmonella* Oslo isolated from seafood and its auxotrophic mutant LAT9. Determination of anti-cancer activity of this laboratory generated auxotrophic mutant using cell line and animal models would provide a suitable alternative to *S*. Typhimurium VNP20009 as candidate strains for bacteria-mediated anticancer therapy.

The whole genome shotgun projects have been submitted to GenBank and the assigned accession numbers are as follows: NZ_SJXK00000000 for SO1 and NZ_SMLR00000000 for LAT9. The version described in the paper represents the first version.
